# Supplementary material for: Effect of Trichoderma asperellum on Wheat Plants’ Biochemical and Molecular Responses, and Yield under Different Water Stress Conditions
Source: Int J Mol Sci. 2022 Jun 17;23(12):6782. doi: 10.3390/ijms23126782 (PMC9224292; doi:10.3390/ijms23126782)
Supplement: Supplementary file 1 [file ijms-23-06782-s001.zip › ijms-1747317-supplementary.pdf]

Table S1. Measurements of macro- and microelements contents on wheat grains of 28-week- old plants.

	**Optimal irrigation**		**Water stress**	
	**Control**	**T140**	**Control**	**T140**
%C	45.49±0.15 a	45.20±0.20 ab	44.97±0.15 b	45.23±0.20 ab
%N	1.45±0.15 b	1.60±0.11 ab	1.63±0.10 ab	1.67±0.09 a
Ca	0.18±0.02 ab	0.19±0.02 a	0.13±0.01 c	0.15±0.01 bc
K	4.83±0.16 a	4.87±0.33 a	5.24±0.20 a	5.02±0.35 a
Mg	1.48±0.11 a	1.56±0.06 a	1.48±0.03 a	1.53±0.05 a
P	4.16±0.33 a	4.53±0.18 a	4.48±0.17 a	4.54±0.14 a
S	1.04±0.09 b	1.15±0.05 a	1.15±0.05 a	1.23±0.04 a
B	2.02±0.15 a	1.57±0.17 b	1.66±0.10 b	1.32±0.15 c
Cu	3.73±0.39 b	4.24±0.32 b	4.38±0.37 b	5.27±0.33 a
Fe	24.30±2.56 b	37.89±22.63 a	24.06±5.45 b	39.10±11.55 a
Mn	60.79±6.98 b	67.80±6.34 ab	73.09±3.01 ab	75.09±3.34 a
Mo	1.97±0.17 a	1.79±0.21 a	1.73±0.28 a	1.67±0.32 a
Zn	30.01±1.55 a	31.3±1.45 a	34.37±4.87 a	34.16±3.93 a

C and N = %; Ca, K, Mg, P, and S = g/kg of grain; Cu, Fe, Mn, Mo, and Zn = mg/kg of grain. Data are the mean of five samples for each treatment and irrigation condition (n = 5). Values in the same row with different letter are significantly different according to Tukey's test ($p < 0.05$).

Table S2. Primers used for real-time quantitative PCR (qPCR) analysis.

Name	Sequences (5′ → 3′)	Hit description	Accession Number	References
TaNAC2-Fw	CTGGGTGCTCTGCCGGCTCTA	NAC2 transcription factor	KY461012	This work
TaNAC2-Rv	CTCCGCCTTGGGCTCCATCATC			
TaDREB2-Fw	CTCTGAAACGATCAGGCGATGG	Dehydration-responsive element-binding protein 2 transcription factor	AB193608	[59]
TaDREB2-Rv	GTGTATTCTCAGGTCCTCCTTTCC			
TaDHN16-Fw	TACGGACAGCAAGGTCATAC	Dehydrin 16 gene	X78429	This work
TaDHN16-Rv	TCCATGATGCCCTTCTTCTC			
TaP5CR-Fw	TGGCTGATGGTGGAGTTG	Pyrroline-5-carboxylate reductase	AY880317	This work
TaP5CR-Rv	GCTGCCCTGGATGTTTAC			
TaNIA7-Fw	CTCAAGCGCAGCACGTCTA	NADH-nitrate reductase	XM_044561748	[48]
TaNIA7-Rv	CTCGGACATGGTGAACTGCT			
TaGS2-Fw	GATGGAGGTTTCGACGTGAT	Chloroplastic glutamine-synthetase	DQ124212	[59])
TaGS2-Rv	CAAGTCATGGCGAAGTGAAA			
TaPDR1-Fw	GAGCAGTATCGTCACGTGTCTGT	PDR-type ABC transporter	FJ858380	[48]
TaPDR1-Rv	TCTGCTGGCCTACGTGGAA			
TaAMT3.1-Fw	TGGAACGTGGTGGTCACCAGC	Ammonium transporter 3 member 1-like	XM_044502734	This work
TaAMT3.1-Rv	AGAGCGCGTAGGCCTCCTCG			
TaUBQ-Fw	GCACCTTGGCGGACTACAACATTC	Ubiquitin (housekeeping gene)	X56803	This work
TaUBQ-Rv	ACACCGAAGACGAGACTTGTGAACC			

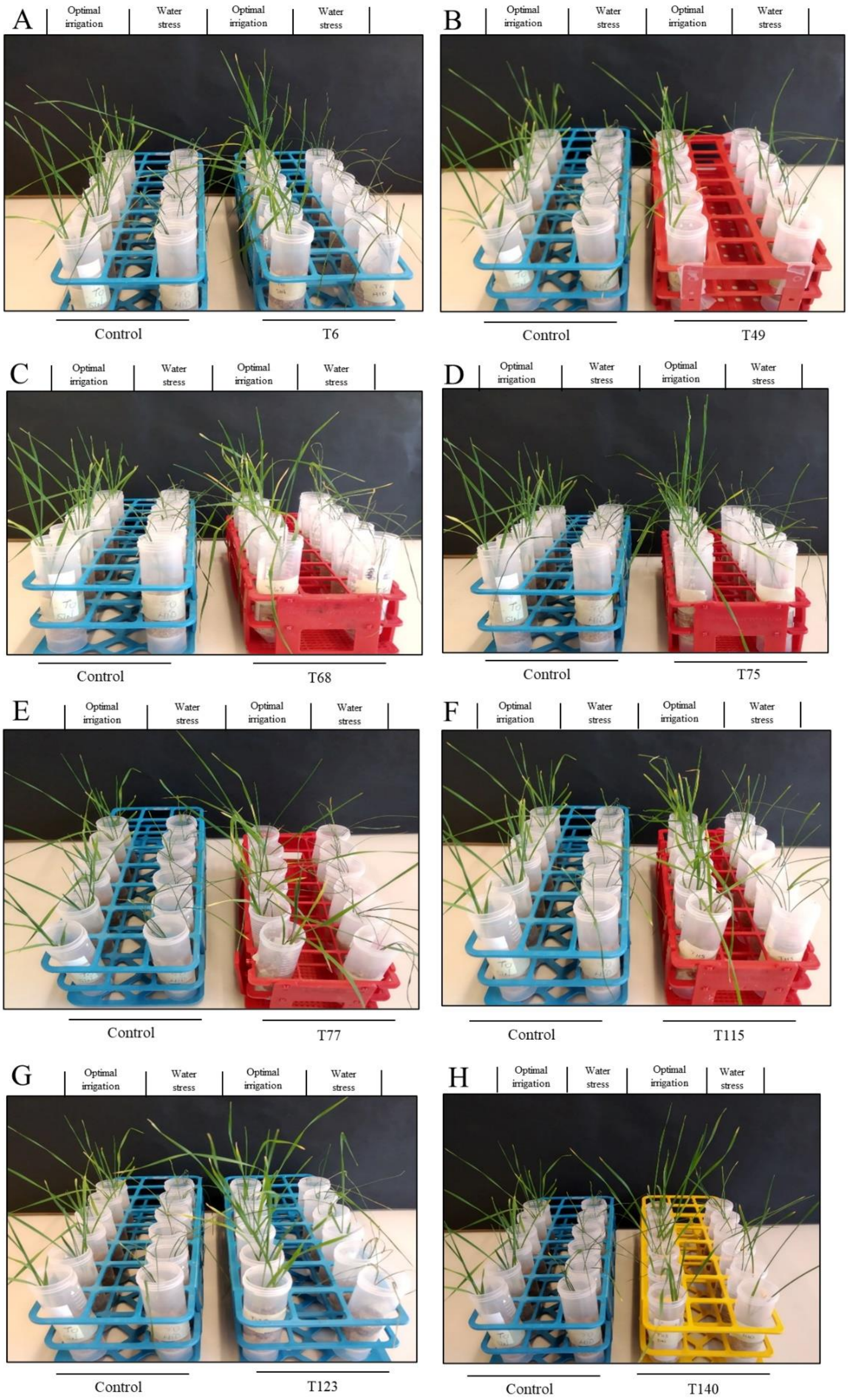

Figure S1. Four-week-old wheat seedlings treated with *Trichoderma* spp. and their respective untreated plants (control) subjected to optimal irrigation or water stress (irrigation withdrawn during the third and fourth weeks).

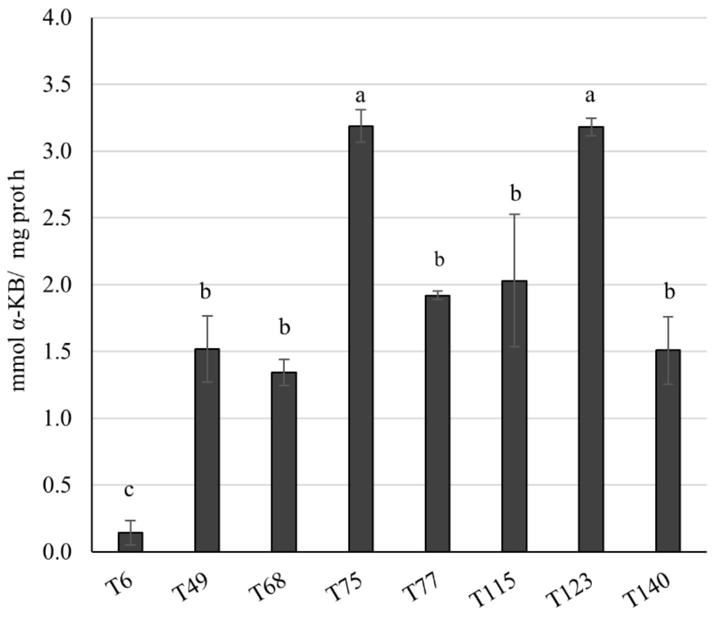

Figure S2. ACCD activity in eight *Trichoderma* strains after being grown at 180 rpm and 28°C for 4 days in synthetic minimal medium.
